# Promyelocytic Leukemia (PML) Protein Plays Important Roles in Regulating Cell Adhesion, Morphology, Proliferation and Migration

**DOI:** 10.1371/journal.pone.0059477

**Published:** 2013-03-21

**Authors:** Mei Kuen Tang, Yong Jia Liang, John Yeuk Hon Chan, Sing Wan Wong, Elve Chen, Yao Yao, Jingyi Gan, Lihai Xiao, Hin Cheung Leung, Hsiang Fu Kung, Hua Wang, Kenneth Ka Ho Lee

**Affiliations:** 1 Stem Cell and Regeneration Thematic Research Programme, School of Biomedical Sciences, Chinese University of Hong Kong, Shatin, N.T., Hong Kong; 2 Joint JNU-CUHK Key Laboratories for Regenerative Medicine, Ministry of Education, JiNan University, Guangzhou, China; 3 Division of Infectious Diseases, School of Public Health and Primary Care, Chinese University of Hong Kong, Shatin, N.T., Hong Kong; University of Birmingham, United Kingdom

## Abstract

PML protein plays important roles in regulating cellular homeostasis. It forms PML nuclear bodies (PML-NBs) that act like nuclear relay stations and participate in many cellular functions. In this study, we have examined the proteome of mouse embryonic fibroblasts (MEFs) derived from normal (PML^+/+^) and PML knockout (PML^−/−^) mice. The aim was to identify proteins that were differentially expressed when MEFs were incapable of producing PML. Using comparative proteomics, total protein were extracted from PML^−/−^ and PML^+/+^ MEFs, resolved by two dimensional electrophoresis (2-DE) gels and the differentially expressed proteins identified by LC-ESI-MS/MS. Nine proteins (PML, NDRG1, CACYBP, CFL1, RSU1, TRIO, CTRO, ANXA4 and UBE2M) were determined to be down-regulated in PML^−/−^ MEFs. In contrast, ten proteins (CIAPIN1, FAM50A, SUMO2 HSPB1 NSFL1C, PCBP2, YWHAG, STMN1, TPD52L2 and PDAP1) were found up-regulated. Many of these differentially expressed proteins play crucial roles in cell adhesion, migration, morphology and cytokinesis. The protein profiles explain why PML^−/−^ and PML^+/+^ MEFs were morphologically different. In addition, we demonstrated PML^−/−^ MEFs were less adhesive, proliferated more extensively and migrated significantly slower than PML^+/+^ MEFs. NDRG1, a protein that was down-regulated in PML^−/−^ MEFs, was selected for further investigation. We determined that silencing *NDRG1*expression in PML^+/+^ MEFs increased cell proliferation and inhibited *PML* expression. Since NDRG expression was suppressed in PML^−/−^ MEFs, this may explain why these cells proliferate more extensively than PML^+/+^ MEFs. Furthermore, silencing *NDRG1*expression also impaired TGF-β1 signaling by inhibiting SMAD3 phosphorylation.

## Introduction

PML protein has been extensively studied because of its involvement in the etiology of Acute Promyelocytic Leukemia (APL) [1]. Approximately 95% of human APL contains the homologous recombination between chromosome 15 and 17 [2]. It involves a translocation between the PML gene in the breakpoint region on chromosome 15q22 and the partner gene Retinoic Acid Receptor alpha (RARα) located on chromosome 17q21. The chimeric proteins formed, PML-RARα and/or RARα-PML, are thought to be responsible for activating the oncogenic events associated with the transformation to the APL phenotype [3]. PML is one of the key components involved in the regulation of numerous important biological functions through its ability to inhibit the ubiquitination and proteasomal degradation processes. PML monitors the activation of p53/TP53 via phosphorylation in the nucleolus following DNA damage and participates in neoangiogenesis and tumor vascularization. It also plays an important role in regulating gene transcription, and other nuclear events, including repression of cell-cycle progression, regulation of cellular senescence, cell death and neurodevelopment [4, 5, 6 & 7]. It has been reported that pRB, p53, mTOR, cJun, Akt, Daxx and EIf4e are all capable of interacting with PML to regulate homeostasis in immune response, to repress cell proliferation and to regulate protein synthesis [8 & 9]. Nevertheless, it is still unclear whether PML is capable of interacting with other proteins during cellular processes.

PML is a Ringer-finger protein that belongs to the tripartite motif family of proteins [10]. In human, there are seven splice variants of PML and all of them perform a variety of distinct functions [7]. Recently, it has been demonstrated that PML variant II is one of the most abundant and plays a key role in regulating the function and structure of PML-NBs [11]. In this study, we have used comparative proteomics to elucidate all the proteins that are differentially expressed in MEFs that are incapable of expressing PML [12]. In addition, we have also examined how the absence of PML affected cell adhesion, morphology, proliferation, migration and gene expression.

## Results

### Comparison of PML^+/+^ and PML^−/−^ MEFs Morphology

**Figure 1:**
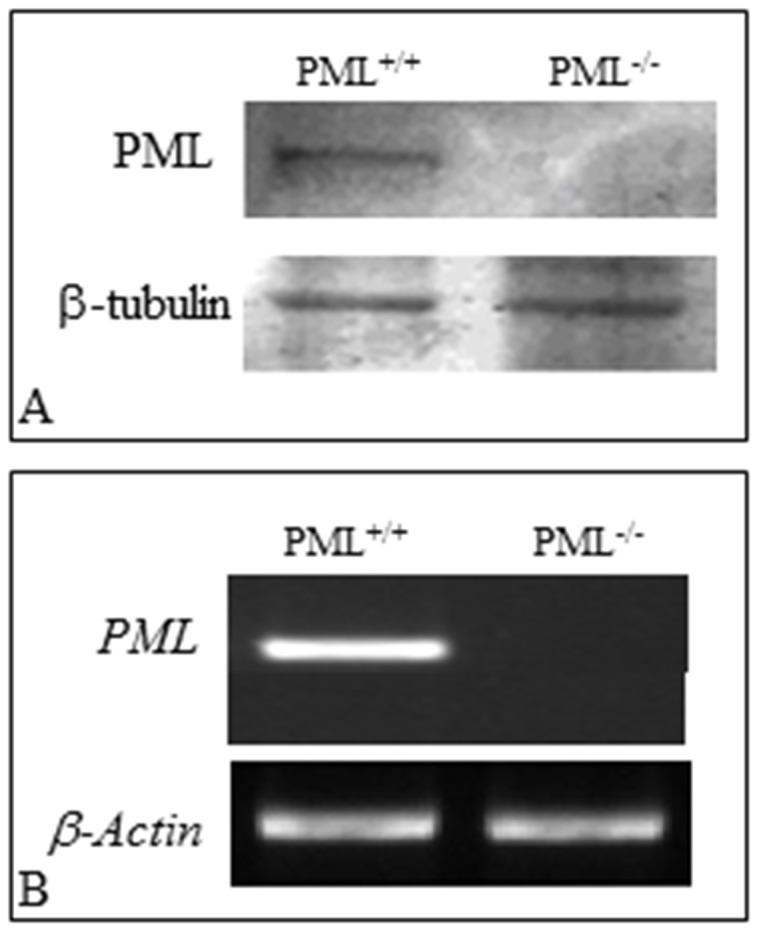
Validation of PML^−/−^ and PML^+/+^ MEFs. (A) Western blot showing PML^−/−^ MEFs do not express PML protein. (B) Semi-quantitative RT-PCR also indicated that PML^−/−^ MEFs do not express *PML*. β-actin served as an internal control.

**Figure 2:**
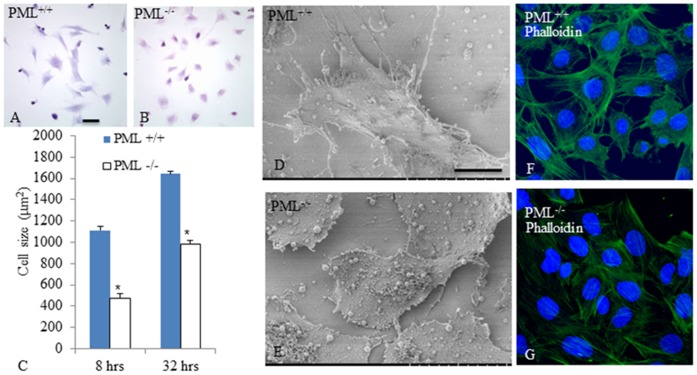
PML^−/−^ MEFs are morphologically different from PML^+/+^ MEFs. H&E staining showing the size of PML^+/+^ MEFs during culture were larger (A) than PML^−/−^ MEFs (B). The average size of PML^+/+^ and PML^−/−^ MEFs were measured 8 hr and 32 hr after culture. The bar chart shows the average size of PML^+/+^ MEFs was significantly larger than PML^−/−^ MEFs. Data are displayed as mean ±SD and analyzed by t-test. *p<0.05 (C). Scanning electron microscopy revealed that the edges of PML^+/+^ MEFs contained numerous elongated membrane projections (D). These cytoplasmic projections were not found on the cell surface of PML^+/+^ MEFs (E). Phalloidin staining was used to demonstrate the actin network. The PML^−/−^ MEFs (G) were not staining as intensely as PML^+/+^ MEFs (F) suggesting that their actin network was not as well developed. Scale bar = 25 µm.

We first confirmed the status of PML^+/+^ and PML^−/−^ MEFs by Western blotting and RT-PCR. The results confirmed that the PML^−/−^ MEFs did not express PML transcripts and protein (Figs. 1A & B). We then estimated the average size of PML^+/+^MEFs, 8 and 32 hrs after culture, to be 1.1±0.03 and 1.6±0.043 mm^2^, respectively. For PML^−/−^ MEFs, the average size after 8 and 32 hr incubations were 0.48±0.023 and 0.98±0.036 mm^2^, respectively (Fig. 2C). The results indicated that the PML^−/−^ MEFs were significantly smaller than PML^+/+^ MEFs. Hematoxylin and eosin staining showed that there was no obvious morphological differences between PML^+/+^ (Fig. 2A) and PML^−/−^ (Fig. 2B) MEFs under the light microscope. However, under the SEM, the cell surface of PML^+/+^ MEFs were found to contain distinct elongated membranous projections. These projections irradiated from the edges of the cells and were attached on the culture plate (Fig. 2D). Similar membrane projections were not found on the cell surface of the PML^−/−^ MEFs; instead, the cell surface was covered by extracellular matrix proteins (Fig. 2E). We also cultured both MEFs on gelatin-coated culture dishes and then stained the MEFs with Phalloidin dye to show the arrangement of the cytoskeleton. Phalloidin staining revealed that filamentous actin was highly organized in both types of MEFs. However, the staining intensity that we produced from PML^−/−^ MEFs (Fig. 2F) were always weaker than PML^+/+^MEFs (Fig. 2G). This may be attributed to PML^−/−^ MEFs containing fewer filamentous actin.

### Differences in Cellular Adhesions between PML^+/+^ and PML^−/−^ MEFs

**Figure 3:**
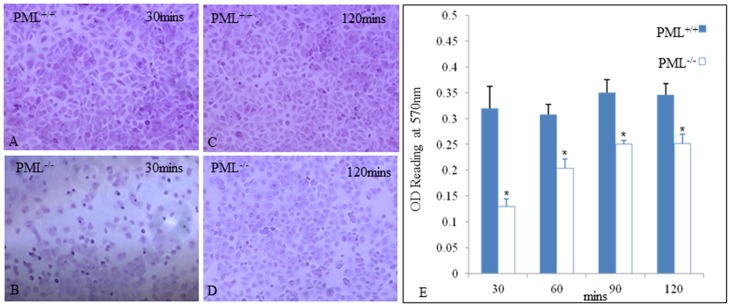
PML^−/−^ MEFs are less adhesive than PML^+/+^ MEFs. The extent of PML^+/+^ (A & C) and PML^−/−^ (B & D) MEFs were able to adhere to the plastic culture dishes, 30–120 min after seeding, was quantitated. (E) Bar chart showing fewer PML^−/−^ MEFs were able to adhere to dish than PML^+/+^ MEFs at all-time point examined.

We measured the efficacy of PML^+/+^ and PML^−/−^ MEFs to adhere to plastic culture dishes 30, 60, 90 and 120 mins after plating. The aim was to establish whether there were any differences in the cell adhesion properties between PML^+/+^ and PML^−/−^ MEFs. We established that there were significantly fewer PML^−/−^ MEFs adhering to the culture dish than PML^+/+^ MEFs, when examined, 30 and 60 mins after plating. The PML^−/−^ MEFs required at least 90 min for cells to fully coat the entire surface of the plastic dish compared with 30 min for PML^+/+^ MEFs (Figs. 3A–D). We also stained the MEFs, after different time intervals of plating, with 0.25% crystal violet dye and then extracted the dye for spectrophotometery to quantify the extent of cell adhesion. The results revealed that at all time points examined, there were significantly fewer PML^−/−^ MEFs adhering to the culture wells than PML^+/+^ MEFs (Fig. 3E).

### Effects of PML Null Mutation on Cell Proliferation

**Figure 4:**
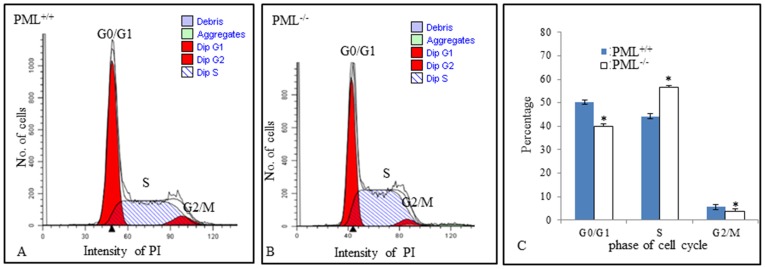
PML^−/−^ MEFs proliferate significantly faster that PML^+/+^ MEFs. Cell cycle analysis of PML^+/+^ (A) and PML^−/−^ (B) MEFs after 32 hr culture. Propidium iodide staining was performed and samples were analyzed by flow cytometry. (A) ModFit analysis revealed that 50.50±0.3% of PML^+/+^ MEFs were distributed at G0/G1 phase, 44.25±0.65% at S phase and 5.52±0.24% at G2/M phase. (B) For PML^−/−^ MEFs, 40.21±0.37% were distributed at G0/G1 phase, 56.43±0.28% at S phase and 3.36±0.17% at G2/M phase. (C) Bar chart comparing the cell cycle profile of PML^+/+^ and PML^−/−^ MEFs, 8 and 32 hr after culture. The experiment was repeated in triplicate. The data are displayed as mean ± SD and analyzed by t-test, *p<0.05.

We investigated whether there were any differences between the cell cycle profile of PML^−/−^ and PML^+/+^ MEFs. All cells were cultured for 36 hours and then dissociated from the culture flasks. They were stained with PI dye, sorted in a FACSAria II Flow Cytometer and analyzed using a ModFit LT^TM^software. We established that there were approximately 12±3.3% more PML^−/−^ MEFs distributed at S-phase than the control PML^+/+^ MEFs (Figs. 4A & B). However, there were 10.3±3.6% and 1.9±0.58% less PML^−/−^ MEFs distributed at G0/G1 and G2/M phases respectively than PML^+/+^ MEFs (Fig. 4C). The results suggest that PML^−/−^ MEFs proliferate significantly faster that PML^+/+^ MEFs.

### Effects of PML Null Mutation on Cell Migration

**Figure 5:**
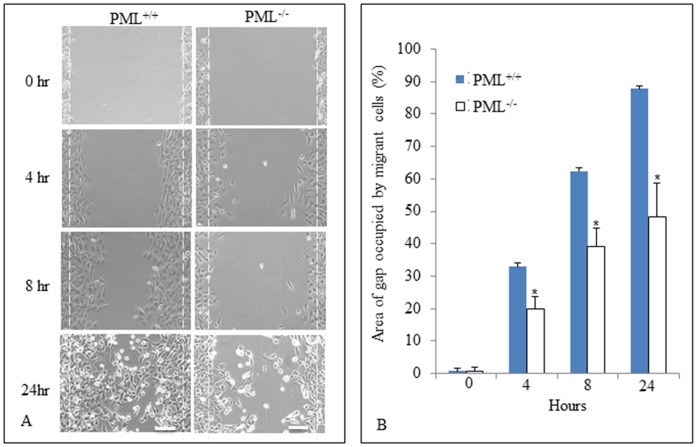
PML^+/+^ MEFs are significantly more mobile than PML^−/−^ MEFs. (A) In vitro scratch migration assay showing the extent that PML^+/+^ and PML^−/−^ MEFs were able to migrate into the gap/space (defined by the dotted white lines) at different time intervals: 0, 4, 8 and 24 hrs. Scale bar = 500 µm. (B) Bar chart showing the percentage area of the gap that have been invaded by PML^+/+^ and PML^−/−^ MEFs, at different time intervals. The experiment was repeated three times. Data is presented as Mean ± SD by t-test and *p<0.05.

The scratch assay was used to assess whether the migration efficiency of PML^+/+^ and PML^−/−^ MEFs were different because of the null mutation. PML^+/+^ and PML^−/−^ MEFs were allowed to grow until confluent and then prevented from further proliferation by Mitomycin C treatment. A scratched (2.5 mm wide) was then created longitudinally in the monolayer culture. Micrographs were taken of these cultures at different time intervals. Two dotted lines were drawn on the plastic dish to define the original width of the gap created in the cultures. The total numbers of MEFs that have migrated into the gap (i.e. in front of the dotted lines) were counted at different time intervals for both PML^+/+^ and PML^−/−^ MEFs (Fig. 5A). The results showed that there were significantly more PML^+/+^ MEFs that have migrated into the gap than PML^−/−^ MEFs at all-time intervals examined. The percentage of migrant cells in the gap at different time intervals was calculated as shown in Figure 5B. After 24 hr culture, 87.7±6.9% area of the gap was filled up with PML^+/+^ MEFs versus 48.3±15% for PML^−/−^ MEFs. The results imply that PML^+/+^ MEFs were significantly more mobile than PML^−/−^ MEFs *in vitro*.

### Differences in the Proteomes of PML^−/−^ and PML^+/+^ MEFs

**Figure 6:**
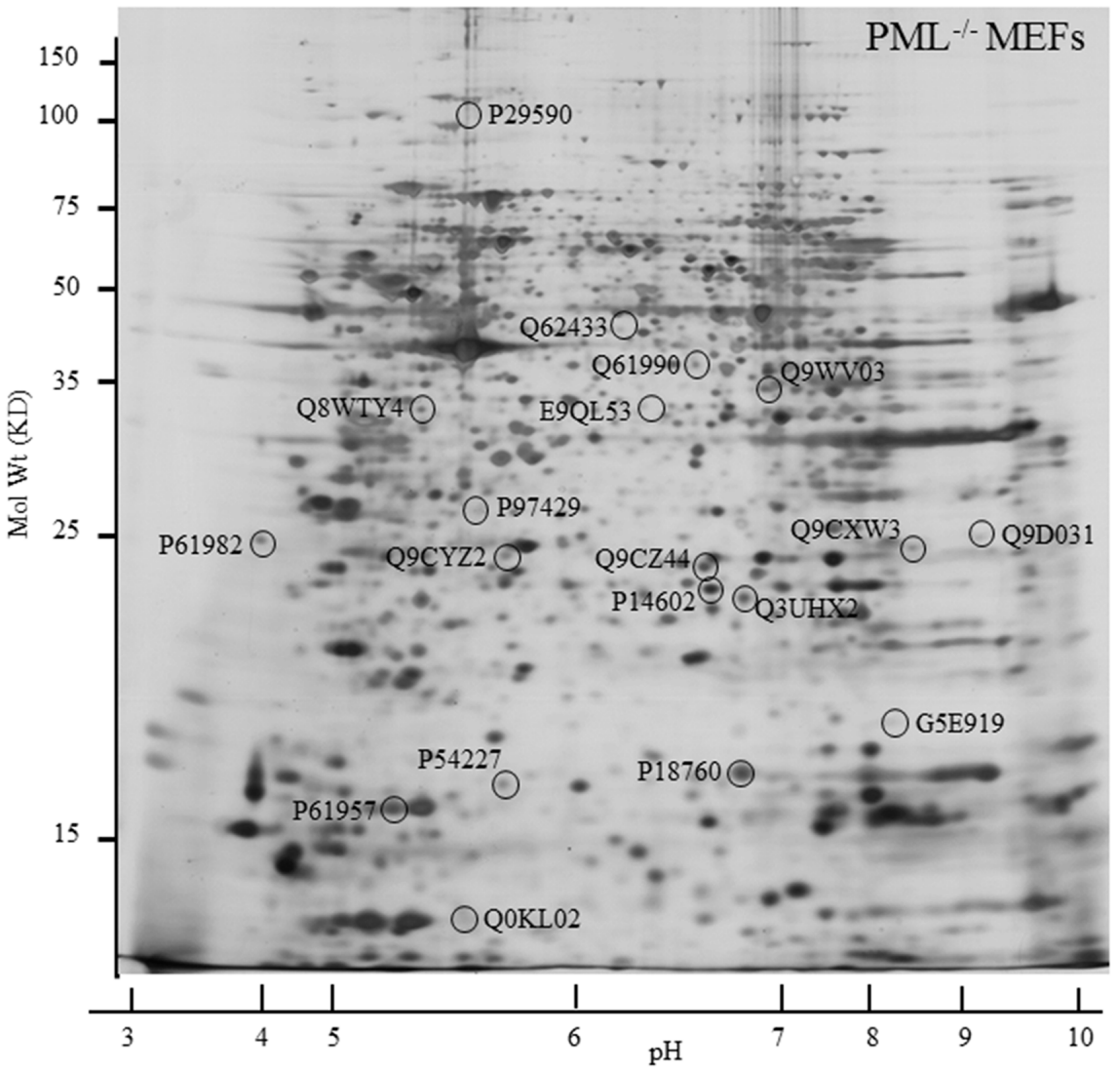
The proteome of PML^−/−^ and PML^+/+^ MEFs are different. Proteins spots in the silver stained 2-DE gel that have been circled were determined to be differentially expressed when 2-DE of PML^−/−^ and PML^+/+^ MEFs were compared. The experiment was repeated in triplicate.

**Figure 7:**
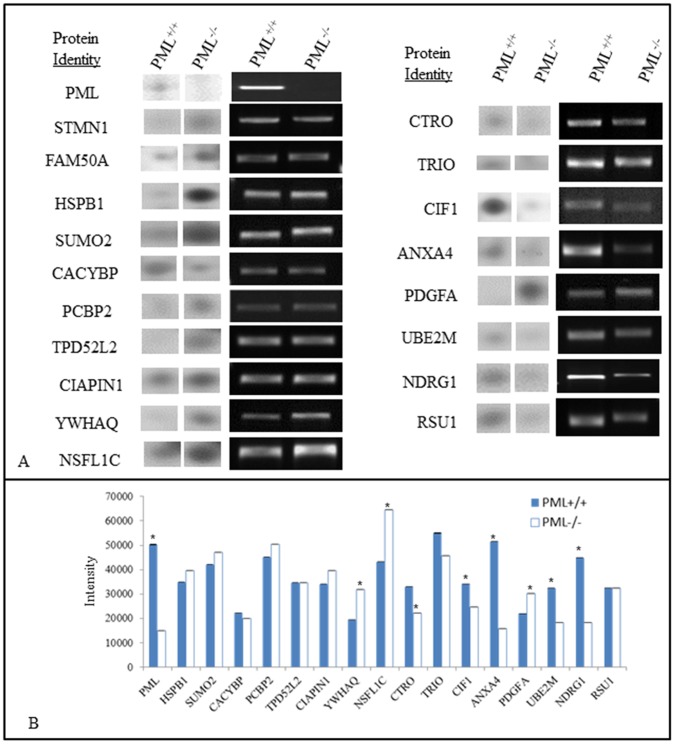
Proteins that are differentially expressed may not be correspondingly reflected at the transcriptional level. (A) LC-ESI-MS/MS identified proteins that were differentially expressed in PML^−/−^ and PML^+/+^ MEFs. Semi-quantitative RT-PCR was performed to establish whether the proteins that we identified were differentially expressed were also correspondingly expressed at the mRNA level. (B) The bar chart shows the intensity of the PCR bands for each gene in (A). The measurements were normalized against β-actin internal control. The data is presented as Mean ± SD by t-test and *p<0.05. The experiment was repeated in triplicates.

2-DE were performed on total proteins extracted from PML^−/−^ and PML^+/+^ MEFs. The resolved gels were silver stained and compared. Proteins that were determined to be differentially expressed were isolated for LC-ESI-MS/MS analysis. The results revealed 19 proteins that were differentially expressed as shown in Figures 6 & 7. Nine proteins were found down-regulated in PML^−/−^ MEFs which includes: (1) promyelocytic leukemia, (2) N-myc downstream regulated 1 gene (NDRG1), (3) calcyclin binding protein (CACYBP), (4) cofilin 1, non-muscle (CFL1), (5) Ras suppressor protein 1 (RSU1), (6) triple functional domain (PTPRF interacting) (TRIO), (7) citron (CIT), (8) annexin A4 (ANXA4) and (9) ubiquitin-conjugating enzyme E2M (UBE2M). In addition, 10 proteins were determined to be up-regulated in PML^−/−^ MEFs which includes: (1) Cytokine induced apoptosis inhibitor 1 (CIAPIN1), (2) family with sequence similarity 50, member A (FAM50A), (3) SMT3 suppressor of mif two 3 homolog 2 (SUMO2), (4) Heat shock protein 1 (HSPB1), (5) NSFL1 (p97) cofactor (p47) (NSFL1C), (6) poly(rC) binding protein 2 (PCBP2), (7) tyrosine 3-monooxygenase/tryptophan 5-monooxygenase activation protein, gamma polypeptide (YWHAG), (8) stathmin 1 (STMN1), (9) tumor protein D52-like 2 (TPD52L2) and (10) PDGFA associated protein 1 (PDAP1). The reported function and the level of change (folds) of these proteins, between PML^−/−^ and PML^+/+^ MEFs are summarized in Tables 1 and 2. We performed semi-quantified RT-PCR analysis to establish whether the proteins that we identified were differentially expressed were also correspondingly expressed at the mRNA level (Fig. 7A and B). We determined that HSPB1, SUMO2, CACYBP, PCBP2, TPD52L2, CIAPIN1 and RSU1 proteins were differentially expressed between PML^−/−^ and PML^+/+^ MEFs but not at the transcriptional level. This suggests that for these proteins the differences observed were a consequence of post- transcriptional regulation.

**Table 1:** Identification of differentially down- regulated proteins in the 2DE.

Protein Identification	Sequences Coverage(%)	Accession No.	Cellular component	Biological process and molecular functions
N-myc downstream-regulated gene 1 protein (Ndrg1)	14	Q62433	-Cell membrane	-DNA damage response, signal transduction by p53 class mediator
			-Cytoplasm	-Cellular response to hypoxia
			-Cytoskeleton	-Peripheral nervous system myelin maintenance
			-Microtubule	-Positive regulation of spindle checkpoint
			-Nucleus	-Stress-responsive protein involved in hormone responses, cell growth, and differentiation.
			-Recycling endosome membrane	
Citron Rho-interacting kinase (Ctro)	46	E9QL53	-Actin cytoskeleton	-Cytokinesis
			-Ruffle	-Dendrite development
			-Vacuole	-Intracellular signal transduction
				-Mitotic metaphase/anaphase transition
				-Mitotic sister chromatid segregation
Triple functional domain protein (Trio)	39	Q0KL02	-cytoplasm	-Regulation of Rho protein signal transduction
				-Coordination of cell-matrix and cytoskeletal rearrangements for cell migration and cell growth
Cofilin-1 (Clf1)	47	P18760	-Cell membrane	-Cytokinesis
			-Cell projection	-Establishment of cell polarity
			-Cytoplasm	-Negative regulation of cell size
			-Cytoskeleton	-Positive regulation of actin filament depolymerization
			-Nucleus	-Protein phosphorylation
				-Regulation of cell morphogenesis
				-Neural crest cell migration
				-Neural fold formation
NEDD8-conjugating enzyme Ubc12 (Ube2m)	40	G5E919	-Cytoplasm	-Positive regulation of neuron apoptotic process
				-Inhibition of the ligase activity of SCF complexes for suppress tumorigenesis and apoptosis
Annexin A4 (Anax4)	19	P97429	-Apical plasma membrane	-kidney development
				-Act as Calcium/phospholipid-binding protein to promote membrane fusion or regulate exocytosis
Calcyclin-binding protein (Cacybp)	17	Q9CXW3	-Cytoplasm	-Ubl conjugation pathway in calcium-dependent ubiquitination
			-Nucleus	-Participation in the ubiquitin-mediated degradation of beta-catenin
Ras suppressor protein 1 (Rsu)	27	Q9D031	-Cytoplasm	-Regulation of Ras signal transduction pathway, growth inhibition, and nerve-growth factor
				-Differentiation processes

**Table 2:** Identification of differentially up-regulated proteins in the 2DE.

Protein Identification	Sequences Coverage(%)	Accession No.	Cellular components	Biological process and molecular functions
Stathmin (Stmn 1)	40	P54227	-Cytoplasm	-Regulation of the microtubule filament system
			-Cytoskeleton	-Differentiation
			-Microtubule	-Neurogenesis
Small ubiquitin-related modifier 2(Sumo2)	19	P61957	-Nucleus	-Ubl conjugation pathway for cellular processes such as nuclear transport, DNA replication and repair, mitosis and signal transduction
Poly(rC)-binding protein 2(Pcbp2)	24	Q61990	-Cytoplasm	-Negative regulator of cellular antiviral responses mediated by MAVS signaling.
			-Nucleus	
Cytokine-induced apoptosis inhibitor 1 (Ciapin1)	30	Q8WTY4	-Cytoplasm	-Apoptotic process or anti-apoptotic effects
			-Nucleus	-Development of hematopoietic cells
NSFL1 cofactor p47(Nsfl1c)	33	Q9CZ44	-Chromosome	Participation in the fragmentation of Golgi stacks during and after mitosis
Protein XAP-5(Fam50a)	24	Q9WV03	-Nucleus	-Act as a DNA-binding protein or transcriptional factor
Heat shock protein beta-1(Hspb1)	30	P14602	-Cytoplasm	-Stress response
			-Cytoskeleton	-Actin organization
			-Nucleus	
NEDD8-conjugating enzyme Ubc12(Ube2m)	40	G5E919	-Cytoplasm	-Positive regulation of neuron apoptotic process
				-Inhibition of the ligase activity of SCF complexes for suppress tumorigenesis and apoptosis
14-3-3 protein gamma (Ywhang)	29	P61982	-Cytoplasm	-Act as an adapter protein for regulation both general and specialized signaling pathways
Tumor protein D52-like 2(Tpd52l2)	22	Q9CYZ2	-Perinuclear region of cytoplasm	-Act as a regulator of cell proliferation

### NDRG1 Expressions in PML^+/+^ and PML^−/−^ MEFs

**Figure 8:**
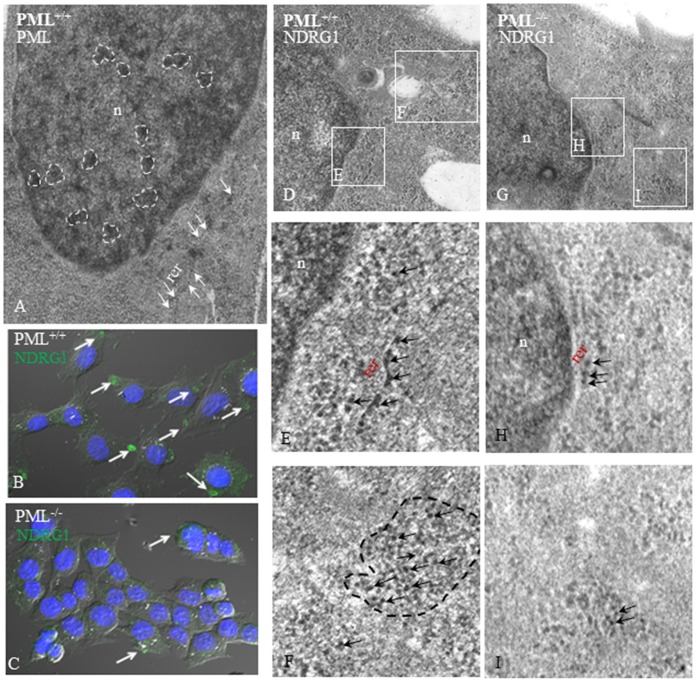
Immune TEM and confocal microscopy showing PML and NDRG1 distribution within MEFs. (A) PML^+/+^ MEFs were stained with PML and QDot conjugated antibodies and then viewed under TEM antibody. PML/QDot labels were found mainly distributed in the rough endoplasmic reticulum (RER) and as numerous small aggregates in the nucleus (white arrows). (B) Immunofluorescent staining revealed that normally NDRG1 was localized as a small oval-shaped aggregate and as speckles in PML^+/+^ MEFs (white arrows). (C) In contrast, there were only small speckles of weakly stained NDRG1 in the cytoplasm of PML^−/−^ MEFs (white arrows). The MEFs were stained with NDRG1 and QDot conjugated antibodies. (D–F) Under TEM, numerous electron dense Qdot labels were discernible in the RER and Golgi complex (black arrows) of PML^+/+^ MEFs. (G–I) For PML^−/−^ MEFs, only a few QDots were evident in the RER and Golgi complex (black arrows).

We have selected one of the proteins (called NDRG1) that were differentially expressed in the proteomic analysis for further investigation. In the proteomics, NDRG1 was expressed in PML^+/+^ MEFs but was barely detectable in PML^−/−^ MEFs. Semi-quantitative RT-PCR was performed and the results correlated with the proteomic findings that there was significantly less NDRG1 transcripts in PML^−/−^ than PML^+/+^ MEFs (Fig. 7). We also examined NDRG1 expression in MEFs using immunofluorescent staining. In PML^+/+^ MEFs, NDRG1 was distributed in the cytoplasm as a small oval-shaped aggregate and as speckles when viewed under the confocal microscope (Fig. 8B). In contrast, there were only small speckles of weakly stained NDRG1 distributed in the cytoplasm of PML^−/−^ MEFs (Fig. 8C). We also examined NDRG1 expression in PML^+/+^ and PML^−/−^ MEFs under the transmission electron microscope (Figs 8D-I). The MEFs were stained with mouse NDRG1 antibody and anti-mouse QDot conjugated secondary antibody. In PML^+/+^ MEFs, there were numerous electron dense Qdot labels present in the rough endoplasmic reticulum (RER) and Golgi complex (Figs. 8D–F). In Golgi complex the QDots were distributed as an aggregate (Fig. 8F) reminiscent of the oval-shaped NDRG1 fluorescent staining detected under the confocal microscopy (Fig. 8B). For PML^−/−^ MEFs, there was a significant reduction in QDot labeling in the RER and Golgi complex (Figs. 8G–I). We also examined the distribution of PML using PML/QDot staining (Fig. 8A). In PML^+/+^ MEFs, PML was distributed mainly in the RER and as numerous small aggregates in the nucleus. Hence, it appears that the distribution of PML and NDRG1 proteins overlap in the RER.

### Differences in Chemotactic Response between PML^+/+^ and PML^−/−^ MEFs

**Figure 9:**
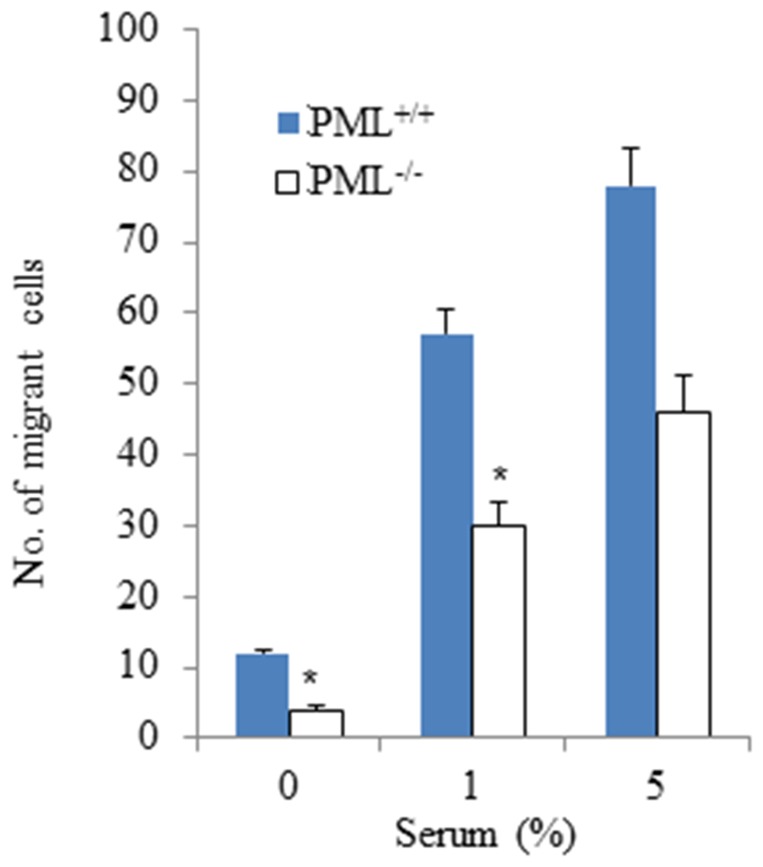
PML^−/−^ MEFs’ migratory chemotactic response is impaired. Bar chart showing the chemotactic migratory response of PML^+/+^ and PML^−/−^ MEFs to different concentration of serum. The experiment was repeated three times. The data is presented as Mean ± SD by t-test and *p<0.05.

The proteomic results revealed that CTRO, TRIO and CLF1 expression were suppressed in PML^−/−^ MEFs. These proteins play an important regulatory role in cell adhesion, spreading and migration. Therefore, we want to establish whether the ability of PML^−/−^ MEFs to chemotactically respond to factors present in fetal bovine serum was affected, when compared with PML^+/+^ MEFs. For this study a Neuro multi-chemotaxis chamber was deployed, where 0, 1% and 5% fetal bovine sera were added to the lower chambers and PML^−/−^ and PML^+/+^ MEFs were added to the upper chambers. Interposed between the upper and lower chambers was a porous sheet of polycarbonate filter through which the MEFs migrated. In the presence of 1% serum and after 4-hour incubation, an average of 57±3.5 of PML^+/+^ MEFs have migrated through the filter compared with 30±0.28 of PML^−/−^ MEFs. For 5% serum, it was 78±5.2 of PML^+/+^ MEFs and 46±3.6 of PML^−/−^ MEFs. The results clearly demonstrated that the PML^−/−^ MEFs’ migratory chemotactic response was deficient (Fig. 9).

### Effects of Silencing *NDRG1* Expression on MEFs Proliferation

**Figure 10:**
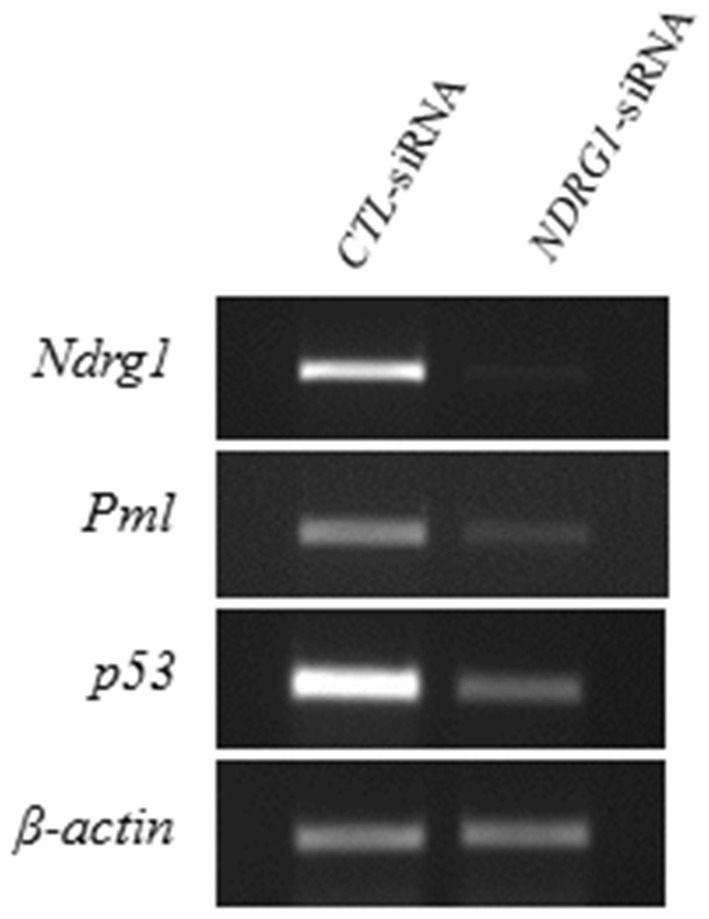
Silencing *NDRG1* in PML^+/+^ MEFs alters gene expression. Semi-quantitative RT-PCR analysis revealed that silencing *NDRG1* inhibited *Ndrg1*, *Pml* and *p53* expression. *β-actin* served as an internal control for normalization.

**Figure 11:**
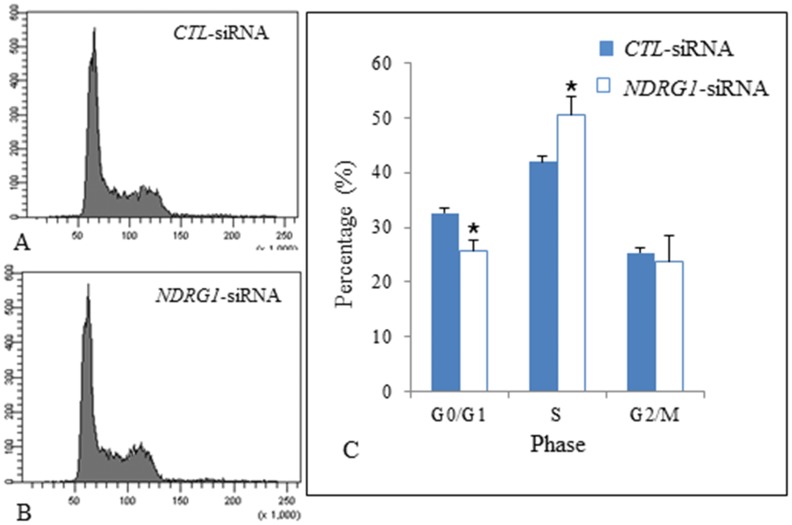
Silencing *NDRG1* expression in PML^+/+^ MEFs increases cell proliferation. (A & B) Cell cycle analysis of PML^+/+^ MEFs transfected with CTL-siRNA and *NDRG1*-siRNA. (C) Bar chart showing that there were significantly more *NDRG1*-silenced MEFs distributed at S-phase than MEFs transfected with *CTL*-siRNAs. Inversely, there were also significantly less *NDRG1*-silenced MEFs distributed in the G0/G1 phase. The data are presented as mean±SD by t-test and *p<0.05.

We have transfected PML^+/+^ MEFs with *CTL*-siRNAs (control) and *NDRG1*-siRNAs for 24 hours and then harvested them for analysis. Semi-quantitative RT-PCR revealed that our *NDRG1*-siRNA was able to silence *NDRG1* expression by approximately 98% (Fig. 10). We also established that *PML* and *p53* expression were also correspondingly inhibited by approximately 95±1.8% and 80±3.6% respectively. Some of these cells were also stained with PI dye and their cell cycle profile analyzed by flow cytometry. We established that for PML^+/+^ MEFs transfected with *CTL*-siRNA, 32.6±3.1% of the cells were distributed at G0/G1 phase, 42.0±3.0% at S phase and 25.3±2.5% at G2/M. For PML^+/+^ MEFs transfected with *NDRG1*-siRNA, 25.7±2.1% of cells were distributed at G0/G1 phase, 50.7±3.2% at S phase and 23.6±4.7% at G2/M phase. The results show that there were significantly more *NDRG1*-silenced MEFs distributed at S-phase than MEFs transfected with *CTL*-siRNAs (Fig. 11). Furthermore, there were significantly less *NDRG1*-silenced MEFs distributed in the G0/G1 phase. The results suggest that silencing *NDRG1* expression increases cell proliferation.

### Effects of Silencing *NDRG1* Expression on TGF-β1 Signaling

**Figure 12:**
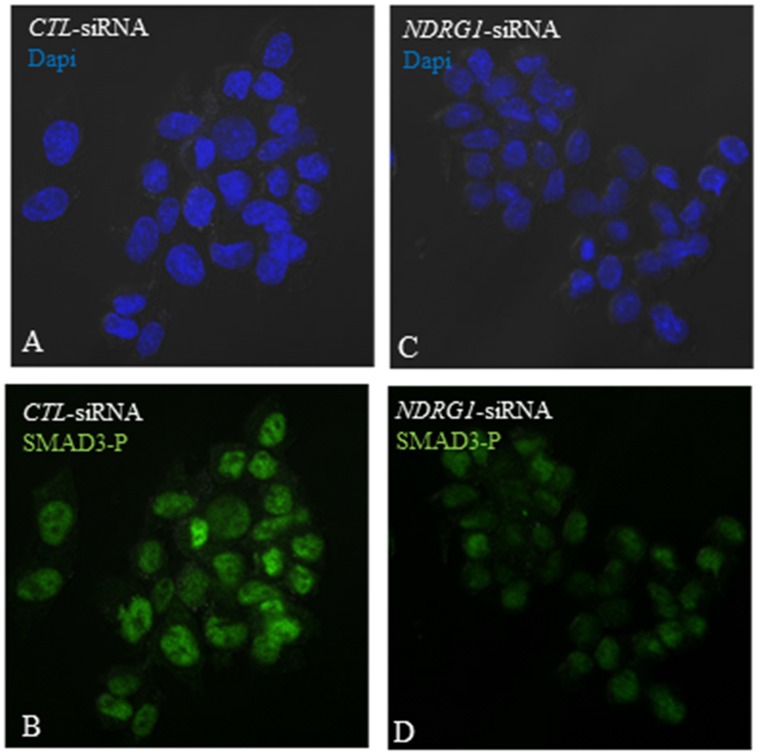
TGF-β1 signaling is impaired in *NDRG1*-silenced MEFs. PML^+/+^ MEFs were transfected with either *CTL*-siRNAs (A & B) or *NDRG1*-siRNAs (C & D) and induced with100 ng/ml TGF-β1. Immunofluorescent staining revealed intense nuclear staining for phosphorylated SMAD3 (SMAD3-P) in *CTL*-siRNA treated MEFs (B) while only weak nuclear staining for MEFs treated with *NDRG1*-siRNA (D).

It has been reported that the TGF-β1 signaling pathway was impaired in PML^−/−^ such that Smad2 and Smad3 phosphorylation was reduced in MEFs [13]. Consequently, this inhibited the nuclear translocation of Smad3 - as the process is phosphorylation-dependent. Presently, our RT-PCR results demonstrated that silencing *NDRG1* expression also inhibited PML expression in PML^+/+^ MEFs. Hence, we wanted to establish whether Smad3 phosphorylation was also correspondingly reduced in *NDRG1*-silenced PML^+/+^ MEFs. We transfected PML^+/+^ MEFs with *CTL*-siRNAs or *NDRG1*-siRNAs for 24 h and then treated the cells with 100 ng/ml TGF-β1. All of the cells were harvested for immunofluorescent staining after 5 hr of TGF-β1 induction. For MEFs transfected with *CTL*-siRNA, TGF-β1 induction resulted in an intense nuclear staining for phosphorylated SMAD3 in all of the cells (Figs. 12A & B). In contrast, for *NDRG1*-silenced MEFs, TGF-β1 treatment only induced weak nuclear phosphorylated SMAD3 staining in approximately 70% of the cells (Figs. 12C & D). The results suggest that TGF-β1 signaling is impaired in *NDRG1*-silenced MEFs.

## Discussions

PML-NBs are sub-nuclear multi-protein structures that have been implicated in many diverse biological functions such as apoptosis, cell proliferation and senescence [14]. In this study, we have used MEFs generated from PML knockout mice to further investigate PML’s biological functions [12]. These MEFs do not express all seven known variants of PML. It has been reported that PML knockout mice react to staphylococcus and listeria infection by hyperproliferative response resulting in the development of splenomegaly [15]. These mice were also very prone to developing spontaneous batryomycosis. Immunohistopathological examinations of the mutant’s lymph nodes, extracted near infection sites, revealed that they were enlarged and hyperplastic. Furthermore, the plasma cells have proliferated dramatically and infiltrated the lesion sites.

We used comparative proteomics to identify proteins that were differentially expressed in PML^−/−^ MEFs. We established that NDRG1 expression was suppressed in the absence of PML. Hence, we decided to examine the relationship between these two proteins. Using immunofluorescent staining, we showed that NDRG1 was distributed as a small oval-shaped aggregate and also as small speckles in the cytoplasm of PML^+/+^ MEFs but only as small speckles in PML^−/−^ MEFs. We further investigated the distribution of NDRG1 using immune TEM. The MEFs were stained with NDRG1and QDot antibodies. Under TEM, we established that the NDRG1-Qdots mainly labeled components distributed around RER and Golgi complex in PML^+/+^ MEFs. Hence, we speculated that NDRG1 was likely involved in the trafficking of endosomes between the RER and Golgi complex for modification and maturation. Furthermore, we also established that PML and NDRG1 were co-located at RER. It has been reported that NDRG1 was normally ubiquitously expressed either in the cytoplasm, nucleus, mitochondrion or cell membrane depending on the tissue type [16]. It appears that NDRG1 is a potential metastatic-related cancer suicide gene because when the gene is over-expressed, it induces apoptosis in human colonic cancer cell lines [17 & 18]. Cellular senescence is regards as an irreversible cell cycle arrest that is associated with the tumor-suppressive mechanism [19 & 20]. Importantly, when we silenced *NDRG1* expression using *NDRG1*-siRNA in PML^+/+^ MEFs, there was a significant increase in cell proliferation. This was also accompanied by an inhibition of PML and p53 expression. It has been reported that the promoter region of NDRG1 contained a p53 binding site and consequently in human colon cancer (DLD-1-p53) cells, NDRG1 is expressed in a p53-dependent fashion following treatment with DNA-damaging chemotherapeutic drug doxorubicin [21]. However, NDRG1 alone was not sufficient for p53-mediated caspase activation and apoptosis.

Lin et al. 2004 has demonstrated that cytoplasmic PML was an essential modulator of TGF-β1signaling [13]. Furthermore, PML^−/−^ MEFs were resistant to TGF-β1 -dependent growth arrest, cellular senescence and apoptosis. TGF-β1 signaling is mediated by the phosphorylation and then nuclear translation of Smad2/3. Cytoplasmic PML can physically interact with Smad2/3 and in its absence Smad2/3 phosphorylation is impaired. In our study, we have shown that silencing *NDRG1* expression in PML^+/+^ MEFs resulted in PML expression being suppressed. Hence, we investigated whether if TGF-β1 signaling was also correspondingly impaired in *NDRG1*-silenced MEFs. Our immunofluorescent staining revealed that phosphorylated Smad3 accumulated in all the nuclei of PML^+/+^ MEFs, 5 hours after TGF-β1 induction. However in *NDRG1*-silenced MEFs, only approximately 70% of the nuclei were weakly stained for phosphorylated Smad3 - while there was no staining in the rest of the nuclei. This suggests that silencing *NDRG1* expression in MEFs impairs the TGF-β1 signaling pathway and this may be mediated by PML because our immune TEM results demonstrated that NDRG1and PML were co-localized in the RER.

Besides NDGR1, our proteomic experiments also identified CTRO, TRIO, ANXA4 and CFL1 peptides were also down-regulated in PML^−/−^ MEFs. These proteins normally play important roles in cell adhesion, spreading and migration. CTRO and TRIO regulate Rho-GTPase to stimulate the cellular responses to changes in cell morphology and directed migration [22 & 23]. Furthermore, ANXA4 has the ability to bind to phospholipids found on membrane surfaces and mediate in the p53-apoptotic pathway [24], enhancement of cancer cell chemoresistance [25], regulates membrane protein mobility [26], membrane trafficking [27] and Ca^2+^ homeostasis [28]. In the developing embryo, CFL1 is involved in regulating cell migration during gastrulation and promoting actin filament assembly [29 & 30]. Bamburg et al. (1999) [31] reported that CFL1 can function as an actin-depolymerizing-factor to destabilize the actin filaments at the leading edge of a migrating cell. In our study, we observed that in the absence of PML the morphology of MEFs was dramatically affected. The PML^−/−^ MEFs were significantly smaller than PML^+/+^ MEFs. Furthermore, PML^−/−^ MEFs did not produce the highly elongated cytoplasmic projections that were so prominent around the cellular edges of PML^+/+^ MEFs as seen under the scanning electron microscope. We believe that these differences in PML^−/−^ MEF morphologies may be attributed to them being less adhesive than PML^+/+^ MEFs (i.e. when cells are highly adhesive they give the appearance of being flatter and therefore larger on culture dishes than less adhesive cell types). Indeed, we have experimentally demonstrated that our PML^−/−^ MEFs were less adhesive than PML^+/+^ MEFs. Besides changes in cell morphology, we have also established that PML^−/−^ MEFs were less mobile and did not respond to chemotactic signals as efficiently as PML^+/+^ MEFs. This again may be attributed to PML^−/−^ MEFs being less adhesive (less traction and therefore less motile) than PML^+/+^ MEFs. These observations raise the possibility that the PML mutation may affect development. Schreck and Gaiano (2009) [32] reported that PML was essential for neural progenitor cells to migrate and develop normally in the neocortex. The brain of PML mutants were shown to be smaller and the neocortical wall thinner than normal animals.

To date, the function of PML is still not fully understood. PML-NBs may act as a nuclear depot for regulating the nucleoplasmic levels of proteins, such as HSF2, SENP-1, PA28, pRb [33, 34, 35 & 36]. They also mediate in nuclear activities (such as repression of p53, Daxx, Mdm2 and Sp100) and act as a nuclear platform for post-translational modification (such as phosphorylation, acetylation, SUMOylation and ubiquitination) [37, 38, 39 & 40]. Interestingly, HSPB1, SUMO2, NSFL1C and STMN1 are proteins that we found up-regulated in our PML^−/−^ MEFs. Most of these proteins are related to the PML-NBs associated proteins, especially to SUMO2 which is involved in SUMOylation [41]. HSPB1 is also known as HSP27 that acts as a chaperone by holding unfolded polypeptides during stress conditions [42]. Inhibition of HSP27 has been reported to induce the degradation of the histone deactylase HDAC6, transcription factor STAT2 and procapase-3 in human cancer cells. PCBP2 expression is up-regulated in PML^−/−^ MEFs. You et al., 2009 [43] reported that PCBP2 was an adaptor of AIP4 which degraded MAVS via the ubiquitin-proteasome pathway. Moreover, PML monitors PCBP2 expression to target proteins for degradation. NSFL1C, another protein that is up-regulated in PML^−/−^ MEFs, is a p47 co-factor that regulates the ATPase activity of membrane fusion protein p97 and leads to Golgi cisternal regrowth [44]. NSFL1C is a substrate of EGF signaling during breast cancer development [45]. It has been demonstrated that antisense-EGFR treatment can increase PML expression in gliobalstoma cells [46]. STMN1 is a 19 kDa cytosolic protein that participates in microtubule-destabilizing in the construction of the mitotic spindle [47]. STMN1 is a biomarker for gastric [48] and non-small cell lung [49] cancers. This protein is normally expressed at low levels but up-regulated in PML^−/−^ MEFs. YWHAG is strongly expressed in PML^−/−^ MEFs and that can interact with RAF1 or miRNA involved in lipid metabolism – hence may inhibit various signal transduction pathways [50 & 51]. Recently, it has been reported that YWHAG is upregulated during osteogenic differentiation [52]. It is not surprising that YWHAG is up-regulated in PML^−/−^ MEFs since PML normally inhibits YWHAG protein to modulate cell proliferation and differentiation [53]. In summary, we have demonstrated that PML plays an important role in cell adhesion, morphology, proliferation, migration and TGF-β1 signaling.

## Materials and Methods

### Cell Cultures

Normal (PML^+/+^) and mutant PML (PML^−/−^) mouse embryonic fibroblasts (MEF) were generously provided by Professor PP Pandolfi [12]. The MEFs were cultured in Dulbecco’s Modified Eagle Medium (DMEM, Life Technology, USA) supplemented with 10% FBS (Life technologies, USA) plus 100 units penicillin and 100 µg streptomycin. The MEFs were maintained at 37°C and 5% CO_2_ in a humidified cell incubator. After the MEFs became confluent, they were trypsinized in 0.25% trypsin solution (Invitrogen, USA) and seeded onto new culture dishes at 1×10^4^ cells/ml.

### Cell Adhesion Analysis

PML^+/+^ and PML^−/−^ (3.5×10^4^ cells/ml) MEFs were plated onto uncoated 96-well plastic culture dish. All un-adhered cells were removed 30, 60, 90 and 120 mins after plating. The wells were gently washed (2X) with warm PBS to remove excess cells and then fixed in 4% formaldehyde for 30 min. The cells were then wash and stained with 0.25% crystal violet dye made up in 40% methanol for 30 mins. After extensive washing to remove residual dye, the cultures were dried and 2% SBS in PBS were added to release the crystal violet dye from the MEFs. The staining intensity was quantified by spectrophotometery (579 nm) using a plate reader. There were 4 replicates for each sample and time point analyzed.

### Western Blot Analysis

Western blot analysis was performed according to methods that we described previously [54]. Briefly, PML^−/−^ and PML^+/+^ MEFs were lysed in lysis buffer (50 mM NaCl, 20 mM Tris, pH 7.6, 1% NP-40, 1X protease inhibitor mixture) for 1 hr. The lysates were centrifuged at 16,000×g at 4°C for 10 min to remove the cell debris and insoluble proteins. Protein concentration was determined by using a Bio-Rad protein assay kit (Bio-Rad, USA). Protein lysate (30–50 µg) from each samples were loaded into the 10% SDS-PAGE for resolving. A Trans-Blot SD semi-dry electrophoretic transfer cell was used to electro-transfer the separated proteins onto Hybond NC membrane (GE Healthcare). The blotted membranes were then blocked with 5% skimmed milk for 1 hr and incubated with either PML (1∶1000, Abcam #53773 or β-tubulin 1∶1000 to 1500, Zymed Laboratories) primary antibodies. Bound antibodies were detected using the appropriate horseradish peroxidase-conjugated secondary antibodies (Southern biotechnology), followed by development with an ECL Western blotting Detection kit (GE Healthcare). The blots were analyzed using Quantity One software (Bio-Rad) and the intensity of the PML stained band was normalized against the β-tubulin band (internal control). Three replicates of each sample were studied.

### Semi-quantitative RT-PCR Analysis

Total RNAs were isolated and purified from PML^−/−^ and PML^+/+^ MEFs in TRIzol solution (Invitrogen Corporation, USA). Using an ImProm-II™ Reverse Transcription System kit (Promega, USA), 1µg of the total RNA was reverse transcribed into complementary DNA (cDNA). Twenty µl of PCR mixture containing 1 µl of cDNA, 2.5 µl of PCR 10X buffer, 0.75 µl of magnesium chloride solution, 1 µl of dNTP mix (10 mM, Promega Corporation, USA), 1 µl of forward primer, 1 µl of reverse primer, 0.25 µl of Taq polymerase (Bio-firm, Hong Kong) and DEPC-treated water in a eppendorf tube was placed into a PTC-100 thermal cycler (MJ Research, Watertown, MA, USA) for PCR amplification. A Primer3 software (version 0.4.0, Rozen and Skaletsky; http://frodo.wi.mit.edu) was used to design all of the primers and determined the PCR amplification conditions as listed in Table 3. Electrophoresis of the PCR products was performed on a 1.5% agarose gel. The produces were stained with GelRed™ dye and the intensities of the PCR product bands were measured using a GelDoc-It imaging system (UVP, BioImaging System, USA). β-actin was used as the internal control and for normalization. The band intensity of each gene expressed was determined and analysed using a MetaMorph® Imaging system software (Molecular Devices, United States). All experiments were repeated three times.

**Table 3:** Primer sequences used in the semi-quantitative RT-PCR Analysis.

Gene Name	Primer Sequences	PCR Conditions
β-actin	Forward: 5′-agcaagagaggtatcctgac-3′	55°C, 20 cycles
	Reverse: 5′-agtaacagtccgcctagaag-3′	
ciapin1	Forward: 5′-aaattgctacctgggtgacg-3′	56°C, 23 cycles
	Reverse: 5′-cacaactgggggagtgactt-3′	
ndrg1	Forward: 5′-ccaaaggcaagaagcagttc-3′	59°C, 24 cycles
	Reverse: 5′-gccaatgctacaaacccagt-3′	
fam50A	Forward: 5′-tgaatgacatgaaggccaaa-3′	56°C, 24 cycles
	Reverse: 5′-ggcatcactgagcagtcgta-3′	
cacybp	Forward: 5′-gatgcaacagaagtcgcaga-3′	56°C, 20 cycles
	Reverse: 5′-ctggggtgctaatgaaggaa-3′	
rsu1	Forward: 5′-ccaccaaatgtagcggaact-3′	59°C, 24 cycles
	Reverse: 5′-gaatgtggagctccttcagc-3′	
trio	Forward: 5′-ctctcgggtggagtcttctg-3′	59°C, 24 cycles
	Reverse: 5′-cctggaaagcacaagagagg-3′	
ctro	Forward: 5′-ggtggtctgtcggagttgtt-3′	60°C, 24 cycles
	Reverse: 5′-taagcacagcctccacctct-3′	
anxa4	Forward: 5′-gcagagattgacatgctgga-3′	59°C, 24 cycles
	Reverse: 5′-tgaggaatgttcagcacgag-3′	
ywahg	Forward: 5′-cagctgagcctacagggaac-3′	56°C, 20 cycles
	Reverse: 5′-agcaaaggtcaaggctgaaa-3′	
stmn1	Forward: 5′-caggtctgttggtgctcaga-3′	60°C, 24 cycles
	Reverse: 5′-agaattgggatcgcaaagtg-3′	
sumo2	Forward: 5′-acgattgatgtgttccagca-3′	56°C, 20 cycles
	Reverse: 5′-acgcttgacttgaagggaaa-3′	
hsbp1	Forward: 5′-ctggggcactcagaaagaag-3′	56°C, 20 cycles
	Reverse: 5′-gctatgcaggcaggtagagg-3′	
clf1	Forward: 5′-ctgctacgaggaggtcaagg-3′	56°C, 20 cycles
	Reverse: 5′-gggatacggagtaggggtgt-3′	
pdap1	Forward: 5′-gatgacaaccagcccagatt-3′	56°C, 20 cycles
	Reverse: 5′-ctttgtgcaaaagcctgaca-3′	
nsf1c	Forward: 5′-accccaagttcagtgtccag-3′	59°C, 24 cycles
	Reverse: 5′-cagccagctctttgttaggg-3′	
pcbp2	Forward: 5′-taccttacggctggtggttc-3′	59°C, 24 cycles
	Reverse: 5′-aaacctgcccaatagccttt-3	
Ube2m	Forward: 5′-gaggacccactgaacaagga-3′	56°C, 20 cycles
	Reverse: 5′-gtcaagagtggggagttgga-3′	

### Immunofluorescent Microscopy

The PML^+/+^ and PML^−/−^ MEFs were fixed in 10% formalin after the 24 hr culture. The samples were then washed with PBS for 10 mins (2X) and treated with 0.5% Triton X-100 for 15 mins to increase cell permeability. To block non-specific binding, the samples were washed with PBS and incubated for 1 hr in 1.5% host serum. The specimens were then incubated in mouse NDRG1 antibody (1∶100, Abcam #ab124689) or SMAD3 (phospho S423+ S425, 1∶100, Abcam # ab52903) overnight at room temperature. After washing with PBS (3X), the specimens were incubated in Cy3-conjugated anti-rabbit (1∶300 dilutions) or anti-mouse QDot conjugated (1∶100, Invitrogen, USA) secondary antibody for 1 hr at room temperature. The samples were further washed with PBS and the nuclei counterstained with 4′, 6-diamidine-2′-phenylindole dihydrochloride (Roche diagnostics, Indianapolis). No primary antibodies were added to the negative control. The immunofluorescently stained PML^+/+^ and PML^−/−^ MEFs were viewed under a BioRad 1024 L Sere Scanning Confocal Microscope (BioRad, USA) equipped with 40 X Zeiss PlanNeofluo objectives.

### Filamentous Actin Staining

The PML^+/+^ and PML^−/−^ MEFs were cultured on round glass coverslips for 24 hr, fixed and washed with PBS. The MEFs were then stained with fluorescein Isothiocyanate labeled phalloidin (1∶100, Sigma #P5282) suspended in 0.5% Triton X-100 and PBS for 15 mins to reveal the presence of filamentous actin inside the cells. After incubation, the samples were rinsed in PBS (3X) for 30 mins and viewed under a BioRad 1024L Sere Scanning Confocal Microscope (BioRad, USA) equipped with 40 X Zeiss PlanNeofluo objectives.

### Immune Transmission Electron Microscopy

PML^+/+^ and PML^−/−^ MEFs were cultured on nylon membrane for 24 hr and washed with 0.2 M cacodylate buffer (0.2 M Sodium cacodylate adjusted to pH 7.2 with HCl). The samples were then fixed in 0.1% glutaraldehyde in 0.2 M cacodylate buffer overnight, washed with 0.2 M Cacodylate buffer (3X) and dehydrated in ethyl alcohol. The dehydrated samples were cleared in propylene oxide, embedded in Epon-812 Medium, sectioned at 50 nm thickness and placed onto lead grids. For immunostaining, the samples were washed in Tris buffer (20 mM Tris, 225 mM NaCl and 20 mM NaN3, adjust to pH8.2 with 0.1 N HCl), blocked with 0.1% BSA in Tris buffer for 30 mins and incubated in rabbit PML (1∶50, Abcam #53773) or mouse NDRG1 antibody (1∶50) overnight. After washing to remove unbound antibodies, anti-mouse QDot conjugated (1∶100, Invitrogen, USA) secondary antibody were added to the samples for 1 hr and then washed with Tris buffer. Finally, the samples were counterstained in 2% uranyl acetate and lead citrate in distilled water for 5 mins. In control samples, primary antibody was not added. The stained samples were examined under a Jeol transmission electron microscope.

### Comparative Proteomic Analysis

First dimensional electrophosis (DE) was performed on an IPGphor IEF system using 11-cm long IPG electrode strip with pH 4–7 gradient (Amersham Biosciences, UK) and an Ettan IPGphor Strip Holder (Amersham Biosciences, UK). 150 µg of PML^+/+^ or PML^−/−^ MEFs protein was applied for each IPG strip. The total volume of protein sample with rehydration buffer (8 M Urea, 2% CHAPS (w/v), 1% IPG buffer (v/v), 40 mM DTT) loaded onto the strip holder was 210 µl. 1 ml of IPG Cover Fluid was applied to each strip so as to minimize evaporation and urea crystallization. The rehydration step was done under voltage and followed by a separation process. The electrophoresis condition for step 1 was 30 V for 13 hrs; step 2 was 500 V for 1 hr; step 3 was 2000 V for 1 hr and step 4 was 5000 V for 20 hrs. The program was stopped when the total volt-hours reached 40000. After the first dimensional DE was completed, the IPG strips were removed from the strip holders. Each strip was then treated with 1% DTT in 6.5 ml of equilibration buffer (50 mM Tris, 6 M of urea, 30% glycerol, 2% SDS, 0.1% bromophenol blue) for 30 min. The medium was then changed to 1% iodoacetamide (IAA, w/v, Sigma-Aldrich, USA) dissolved in the 6.5 ml of the same equilibration buffer. The strips were treated in the solution for 30 min and then loaded onto 12% SDS-polyacrylamide gels with 0.2% agarose in electrophoresis running buffer (25 mM Tris, 192 mM glycine, 0.1%SDS, adjust to pH8.3). Protein markers (20 to 120 kDa, Fermentas Life Sciences) were also loaded into the gel for determining the size of all the proteins resolved in the gel. The 2-DE was performed in an ISO-DALT apparatus (Hoefer Scientific Instruments) at room temperature under constant voltage 100 V till the dye front reached the bottom of the gel. The gels were then fixed in 50% methanol, 12% acetic acid and 0.5 ml 37% formaldehyde for 1 hour. After fixation, the gels were washed in MilliQ water (4X), 50% ethanol (v/v) for 20 min (2X), 0.02% sodium thiosulphate (w/v, Merck, UK) for 10 min and distilled water (3X). Subsequently, the gel was stained in silver solution (0.15% silver nitrate in 0.75 ml 37% formaldehyde) at 4°C for 1 hr. After several brief washes, the gels were developed in developer solution (1 ml 37% formaldehyde, 30 g sodium carbonate and 2 mg sodium thiosulphate in one liter buffer) until the desired staining intensity was attained. The gels were then immersed in 5% acetic acid (v/v, BDH Chemicals Ltd., UK) for 5 mins to terminate the staining process. Finally, the silver stained gels were scanned using a GS 800 Densitometer (Bio-Rad Laboratories, USA) and the images captured were used for image analysis. The protein spots on the gel were analyzed using a PDQuest 2D Analysis Software version 7.13 PC (The Discovery Series, Bio-Rad Laboratories, USA). Each experiment was performed in triplicates.

### Protein Identification by Mass Fingerprinting

All protein spots of interest were isolated from the gel and processed for silver destaining. The gel pieces were first washed in MilliQ water, immersed in 200 µl of destaining solution (15 mM potassium ferricyanide and 50 mM sodium thiosulphate) and then incubated at room temperature until they turned colorless. Each gel pieces was then washed in 400 µl of MilliQ water for 15 min (3X). The destained gel pieces were then equilibrated in 200 µl of 10 mM ammonium bicarbonate/50% acetonitrile for 15 min. The gel was dehydrated in 200 µl of acetonitrile for 15 min and dried at 30°C for 5 min. The gels were digested with 15µg/ml of trypsin in 40 mM ammonium bicarbonate/50% acetonitrile (v/v) at 35°C for 16 hrs. Three µl of extraction solution (50% acetonitrile (v/v) and 5% trifluoroacetic acid (Fluka Chemika, Switzerland) were used to stop the reaction. Three µl of reaction mixture was mixed with α-cyano-4-hydroxycinnamic acid matrix and then spotted onto a sample plate for ESI-MS/MS analysis (Bruker Daltonics, USA). The mass spectrums generated were analyzed using a Bruker Daltonics software and by mass fingerprinting, which were submitted to the SwissPort bioinformation stations using MASCOT 2.2.07 engine search.

### Flow Cytometry

MEFs were stained with Propidium Iodide (PI) dye and processed for flow cytometry as described by Yau et al., 2010 [55]. Briefly, the MEF cultures were trypsinized, suspended and fixed in 70% ethanol overnight at 4°C. The samples were then washed with PBS (2x) and incubated in 20 µg/ml PI (Sigma, USA), 0.1% Trixton X-100 (Sigma, USA) and 100 µg/ml RNase A (Sigma, USA) in the dark for 1 hr. The DNA content analysis was performed in a BD FACSAria II Flow Cytometer ((Becton-Dickinson, Mountain View, CA) and the cell cycle profile was established using a ModFit LT™ software (Verity Software House, USA).

### Scanning Electron Microscopy

Morphology of PML^−/−^ and PML^+/+^ MEFs were examined under the scanning electron microscope (SEM). Briefly, the cells were cultured on 13 mm^2^ round glass coverslip for 24 hr and then fixed in 4% paraformaldehyde and 2.5% glutaldehyde for 24 hrs. The cultures were washed with PBS (3X) and treated with 1% Osmium tetroxide in 0.1 M phosphate buffer. After washing with PBS, the specimens were dehydrated, critical point dried and coated with Gold. The cells were viewed under a SU6600 Variable Pressure Schottky FE-SEM (Hitachi Technologies, Japan).

### In vitro Scratch Assay

The scratch assay was performed according to methods described by Liang et al. [56]. PML^+/+^ and PML^−/−^ MEFs (0.5 ml of 1×10^5^ cells/ml) were first seeded onto 4-well culture plates. After 16 hr incubation, the cells formed a monolayer with 80% confluence. Before a gap was created in the culture, the cells were treated with 2 µg/ml mitomycin C (Sigma, USA) for 1 hr to prevent further cell proliferation (which could confound our interpretation of the cell migration analysis). After the treatment, a sterile p10 pipet tip was used to scrap off cells from the center of the monolayer to create a gap in the culture. Reference lines were etched onto the bottom of the plastic culture dishes to define the position of the gap/wound. Photos were taken of the PML^+/+^ and PML^−/−^ MEFs migrating into the wound area at 0, 4, 8 and 24 hr incubation. The photographic images captured were quantitatively analyzed by establishing the number of cells that have migrated into the gap.

### Chemotactic Assay

Chemotactic cell migration assay was performed according to modified methods described by Webb and Lee [57]. A Neuro multi-chemotaxis chamber (Neuro Probe, Inc., Gaithersbury, USA) consisting of a sheet of porous polycarbonate membrane (25×80 mm with 8.0 µm pores) sandwich between a series of upper and lower wells was used to perform the assay. An appropriate volume of DMEM medium (control), DMEM +0.5% FBS and DMEM +10% FBS were introduced into the lower wells. In the top wells were added 1×10^5^ cells/ml PML^−/−^ or PML^+/+^ MEFs (suspended in DMEM). The multi-chemotaxis chamber was then incubated at 37°C and 5% CO_2_ for 4 hrs. After incubation, all the cells on the top wells were removed and the upper surface of the membrane wiped clean of all attached cells using a cotton swab. The membrane was then fixed in 10% formalin for 1 hr, stained with Hematoxylin and Eosin dyes and mounted with 10% glycerol. The total number of migratory cells found on the bottom surface of the polycarbonate membrane were determined and statistically analyzed.

### Silencing *NDRG1* Expression in MEFs

A synthetic 21 base-pair long siRNA (Santa Cruz, USA) was used to target and silence *NDRG1* expression. The method used for delivering the *NDRG1*-siRNAs and *CTL*-siRNA (control) into PML^+/+^ MEFs has been described by Tang et al., 2006 [58]. Briefly, 0.25 ml of 1×10^5^ PML^+/+^ MEFs were seeded onto each well of a 4-well culture plate and cultured for 16 hrs until approximately 60% confluent. The culture medium was then replaced with Opti-MEM® I Reduced Serum Medium (Invitrogen, USA) containing 2% FBS for 1 hr. One µl of Lipofectamine® RNAiMAX Transfection Reagent (Invitrogen, USA) and 20 nM of either *NDRG1*- or *CTL*-siRNAs were used for transfection. The MEFs were transfected twice (at 6 hrs intervals) and cultured for 24 hrs. The cells were then collected for semi-quantitative RT-PCR, flow cytometry analysis and immunofluorescent staining.

### Statistics

The data were analyzed using two-tailed, paired student’s t-test. P<0.05 was considered to be statistically significant. All statistical analysis was performed using a SPSS software.
